# Network Pharmacology-Based and Clinically Relevant Prediction of the Potential Targets of Chinese Herbs in Ovarian Cancer Patients

**DOI:** 10.1155/2020/8965459

**Published:** 2020-10-08

**Authors:** Jing Sun, Jinfeng Liu, Dan Liu, Xiongzhi Wu

**Affiliations:** ^1^Tianjin Medical University Cancer Institute and Hospital, National Clinical Research Center for Cancer, 300060 Tianjin, China; ^2^Key Laboratory of Cancer Prevention and Therapy, 300060 Tianjin, China; ^3^Tianjin's Clinical Research Center for China, 300060 Tianjin, China

## Abstract

Reports increasingly suggest that Chinese herbal medicine (CHM) has been used to treat ovarian cancer (OvCa) with a good curative effect; however, the molecular mechanisms underlying CHM are still unclear. In this retrospective study, we explored CHM's molecular targets for the treatment of OvCa based on clinical data and network pharmacology. We used the Kaplan-Meier method and Cox regression analysis to verify the survival rate of 202 patients with CHM-treated OvCa. The association between CHM and survival time was analyzed by bivariate correlation. A target network of CHM active ingredients against OvCa was established via network pharmacology. Cox regression analysis showed that CHM is an independent favorable prognostic factor. The median survival time was 91 months in the CHM group and 65 months in the non-CHM group. The survival time of FIGO stage III patients in the two groups was 91 months and 52 months, and the median survival period of FIOG stage IV patients was 60 months and 22 months, respectively (*p* < 0.001). Correlation analysis demonstrated that 12 herbs were closely associated with prognosis, especially in regard to the long-term benefits. Bioinformatics analysis indicated that the anti-OvCa activity of these 12 herbs occurs mainly through the regulation of apoptosis-related protein expression, which promotes OvCa cell apoptosis and inhibits OvCa development. They also regulate the progress of OvCa treatment by promoting or inhibiting protein expression on the p53 signaling pathway and by inhibiting the NF-*κ*B signaling pathway by directly inhibiting NF-*κ*B.

## 1. Introduction

Globally, ovarian cancer (OvCa) is the seventh most commonly diagnosed cancer among women and the eighth most common cause of cancer death, with a five-year survival rate below 45% [1, 2]. More than 75% of affected women are diagnosed at an advanced stage due to the lack of clear signs or symptoms in the early stages of the disease. Less than one-half of the patients survive for more than 5 years after diagnosis [3, 4]. The prognosis for women with advanced OvCa remains poor, despite improvements in the treatment of the disease through cytoreductive surgery followed by system chemotherapy [5–7].

Chinese herbal medicine (CHM) used to treat cancer can be traced back one thousand years [8]. CHM anticancer activities are reported to be through the induction of cancer cell apoptosis, differentiation, cell cycle arrest, prevention of cancer cell growth, invasion and metastasis, and the inhibition of tumor angiogenesis [9–11]. Currently, CHM treats OvCa by enhancing its sensitivity to carboplain, reversing the epithelial-to-mesenchymal transition, inhibiting cancer cell proliferation, migration and invasion ability, and promoting apoptosis to control OvCa's occurrence and development [12–14]. Unfortunately, CHM's active ingredients and potential targets that are directly related to OvCa treatment remain unclear.

**Figure 1:**
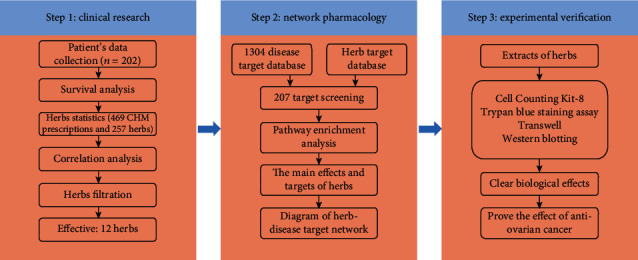
Work process.

With the development of bioinformatics, network pharmacology has been proposed to solve this problem, which is an effective method for studying CHM, as it can systematically and comprehensively reveal CHM's bioactive components and the relationships between potential targets [15–18]. In this article, we evaluated 202 patients with OvCa to determine the efficiency of CHM and unearth the herbs that were strongly correlated with survival. Moreover, we observed the prospective pharmacological mechanisms of CHM using network pharmacology methods. Finally, a series of experiments were conducted to confirm CHM's pharmacological mechanisms against OvCa. The research design flow chart is shown in Figure 1.

## 2. Material and Methods

### 2.1. Statement

Our study did not pose a risk to patients, and did not have negative effects on patients' rights and health. The confidentiality and personal information of the subjects were protected, and the majority of patients had died or were able to be contacted. In accordance with the informed consent exemption rules of the Institute of Cancer and the Tianjin Medical University Hospital, our study complies with the informed consent exemption requirement.

Patients were included in our research according to the following criteria: clear pathological diagnosis of surgery or cytology and CHM treatment in the CHM group ≥2 months. Cases were excluded using the following criteria: serious disease, concurrent cancer, incomplete medical records, lack of accurate documentation of the recurrence time, no distant metastasis, loss to follow-up, and abnormal death. Finally, the medical records of 202 patients diagnosed with OvCa between November 2005 and June 2013 were retrospectively reviewed. Eighty-seven patients who received CHM ≥ 2 months were assigned to the CHM group, and 115 patients who did not receive CHM were included in the non-CHM group.

### 2.2. Treatment

CHM treatment is adopted according to different syndromes. In our clinical studies, formulas for OvCa patients typically contain 15 to 30 herbs. The formula was taken orally 3 times a day, 30 minutes after meals, for 2 months or more in the CHM group. Every 2 weeks, some herbs were changed based on the patient's symptoms. During CHM treatment, patients in the CHM group also received Western medicine (WM) treatments. The patients in the non-CHM group only received WM treatment. The WM treatment included radiotherapy, biotherapy, and targeted therapy.

Patients' formulae were gathered every 2 weeks and counted from the date the CHM treatment was received to the date the patients died or the time of data closure. We analyzed the CHM formulae of 87 patients, who received 469 prescriptions of CHM treatment and 257 types of herbs in total. Among the 257 herbs, some herbs were frequently used, while some herbs were infrequently used. The commonly used herbs were shared among most patients and were closely associated with survival. The infrequently used herbs were often administered to relieve various uncomfortable symptoms, such as abdominal pain, bloating, decreased appetite, fever, or frequent urination. We adopted the use frequency to identify commonly used herbs. The herbs with a frequency > 30 were selected for bivariate correlation analysis. In addition, we calculated the coefficients of correlation between each separate herb and survival time. These herbs were used in a further network pharmacology dissection according to the following criteria: single medicinal substance frequency/maximum value of single herb frequency > 8% and *p* value < 0.05 based on the results of the correlation analysis.

### 2.3. Significant Targets of OvCa

Candidate targets related to OvCa were obtained from the Therapeutic Target Database (TTD) (http://db.idrblab.net/ttd/) and Comparative Toxicogenomics Database (CTD) (http://ctdbase.org/).

### 2.4. Herb Formulation Ingredient Collection, Target Fishing, and Function Scoring

The chemical ingredients of herbs and their predicted targets were obtained from the TCM Systems Pharmacology Database (TCMSP database, http://lsp.nwu.edu.cn/tcmsp.php), Traditional Chinese Medicines Integrated Database (TCMID, http://119.3.41.228:8000/tcmid/), and Bioinformatics Analysis Tool for Molecular mechANism of Traditional Chinese Medicine (BATMAN-TCM, http://bionet.ncpsb.org/batman-tcm/), then selected for further research. The ingredients were screened based on their drug-likeness (DL) value. If the DL ≥ 0.18 (the standard recommended by the TCMSP database), the ingredients were retained. We selected target proteins with scores > 20 (the standard recommended by the BATMAN-TCM) [19].

### 2.5. Network Construction and Analysis

The methods applied for network construction and analysis were similar to those used in our previous studies [20]. Briefly, the ingredient-target networks of herbs and herb-target networks were constructed using the Cytoscape software (Version 3.2.2) and were analyzed using the Cytoscape plugin CentiScaPe. We predicted the main components and targets through calculations of the optimal topological structure and analysis of network statistical properties. Finally, we used the Kyoto Encyclopedia of Genes and Genomes (KEGG) pathway analysis to predict candidate signal pathways.

### 2.6. Preparation of CHM Aqueous Extract

According to the average dosage of the core drugs in clinical, we weighed the drugs as powder and soaked them in deionized water overnight. The material-to-liquid ratio is 1 : 10. After ultrasonic shaking for 1 h, the drug solution was collected and centrifuged to collect the supernatant, then filtered three times with filter paper to remove drug residues. Finally, the filtrates were combined, concentrated in a rotary evaporator to collect the liquid, and stored in a −80°C refrigerator for 48 h. They were then lyophilized for 48 h, and the obtained drug was ground. This is the drug water extract powder. It can be used after being dissolved by concentration.

### 2.7. Cell Culture

SK-O-V3 cell was cultured in 1640 (Invitrogen, Germany) with 10% fetal calf serum at 37°C in a humidified atmosphere of 95% air and 5% CO2. SK-O-V3 was purchased from the Cell Bank of the Chinese Academy of Sciences.

### 2.8. Cell Proliferation Assay

Cell viability and proliferation were examined by trypan blue staining and CCK-8 assays (Sigma). Exponentially grown SK-O-V3 cells (1–2 × 10^4^) were plated in 24-well plates. Following treatment, cells were counted using 0.4% trypan blue to verify viability. SK-O-V3 cells were seeded onto 96-well plates (2000–3000 cells/well), and the CCK-8 kit was added into each well. After incubation, the absorbance values were determined by microplate luminometer (Bio-Rad, USA) at 450 nm.

### 2.9. Transwell Assays

Cell Transwell assays were performed using the Transwell chamber (Corning). After 16 h, cells migrated through the Transwell membrane were fixed with 4% paraformaldehyde and stained with crystal violet. After taking photographs, the number of invaded cells was counted.

### 2.10. Western Blot Analysis

Cell lysate proteins were resolved by 10% sodium dodecyl sulfate polyacrylamide gel electrophoresis (SDS-PAGE) and transferred to PVDF membranes. Each membrane was incubated with monoclonal antibodies against VDR (1 : 2000), BCL-2 (1 : 2000), NF-*κ*B (1 : 2000), p-NF-*κ*B (1 : 1000), TP53 (1 : 2000), p-TP53 (1 : 1000), FAS (1 : 2500), BAX (1 : 2000), and *β*-actin (1 : 2000), followed by incubation with peroxidase-conjugated secondary antibodies and chemiluminescence detection.

### 2.11. Statistical Analysis

The OS was defined as the time from the diagnosis of OvCa to the day of death or the last follow-up of patients with OvCa. The chi-square test and Kaplan-Meier method were used to compare baseline characteristics and testing survival rates, respectively. Prognostic factors were predicted by multivariate Cox regression analyses. The Spearman's bivariate correlation analysis was taken to determine the herbs related to survival. A *p* value of <0.05 was considered statistically significant. Statistical analysis was carried out by SPSS 21.0.

## 3. Results

### 3.1. Patient Characteristics

In the present study, we retrospectively studied 202 patients diagnosed with OvCa at the Tianjin Medical University Cancer Institute and Hospital. A total of 87 patients were treated with CHM, while 115 patients were not treated with CHM. All patients underwent surgical resection. The baselines of patient demographics are summarized in Table 1. Statistical results show that patient age, tumor location (left/right/bilateral), pathology type (endometrioid adenocarcinoma/serous adenocarcinoma/other types), FIGO stage (I-IV), CA-125 Level (<35 kU/L/35 kU/L-280 kU/L/>280 kU/L), chemotherapy method (intravenous injection/intravenous injection+intraperitoneal perfusion), radiotherapy (yes/no), biotherapy (yes/no), and targeted therapy (yes/no) did not differ significantly between the two groups.

### 3.2. Univariate and Multivariate Analyses

Univariate analysis revealed that age (*p* = 0.028), tumor location (*p* = 0.001), FIGO stage (*p* < 0.001), CA-125 (*p* = 0.009), chemotherapy method (*p* = 0.034), radiotherapy (*p* = 0.032), and CHM (*p* < 0.004) were significantly associated with median overall survival (OS).

Patient- and treatment-related variables were significantly associated with overall survival (OS). Cox regression analysis was used to confirm independent risk factors for the survival of patients with OvCa. Case analysis showed that radiotherapy and CHM were protective factors. In our study, after multivariate analysis and calculation, the *β* of CHM was -0.853, and the Exp(*β*) was 0.426 that means CHM was independent protective factor. On the contrary, age (≥60), FIGO stage IV, and CA-125 (>280 kU/L) are risk factors. In addition, patients with bilateral tumors, serous adenocarcinoma, or intravenous+intraperitoneal infusion of chemotherapy face a greater risk of death (Table 2).

### 3.3. Survival Analysis

**Figure 2:**
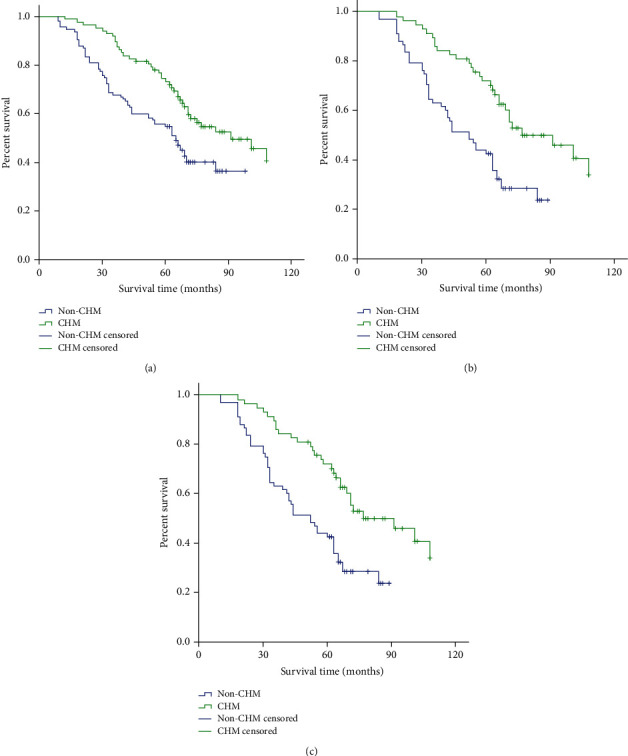
Survival analysis between CHM and non-CHM groups. (a) Patients who received CHM treatment had a longer median OS than those without CHM treatment (91 vs. 65 months, *p* < 0.001). (b) The median survival time of FIGO stage III patients in the two groups was 91 months and 52 months (*p* < 0.001). (c) The median survival period of FIGO stage IV patients was 60 months and 22 months, respectively (*p* < 0.001).

The OS curves for CHM and non-CHM groups are shown in Figure 2. The median survival time was 91 vs. 65 months, and the 2-, 3-,5-, and 7-year OS rates were 96.6%, 89.7%, 70.1%, and 27.6% vs. 80.9%, 67.8%, 54.8%, and 2.6% in the CHM and non-CHM group, respectively. The median survival time of FIGO stage III patients in the two groups was 91 months and 52 months, and the median survival period of FIGO stage IV patients was 60 months and 22 months, respectively. The Log-rank test revealed significant differences between the two groups in terms of OS (*p* < 0.001).

### 3.4. Candidate Targets Associated with OvCa

Despite the fact that there are already hundreds of significant genes and proteins that demonstrate genetic variation in OvCa, only a few major genes and proteins are identified as drug targets. The Therapeutic Target Database (TTD) (http://db.idrblab.net/ttd/) and the Comparative Toxicogenomics Database (CTD) (http://ctdbase.org/) were used to provide information about known and explored therapeutic proteins and nucleic acid targets. We obtained 1304 targets with inference scores above 15 from the two databases.

### 3.5. OvCa-Related Candidate Herbs and Their Putative Main Ingredients and Targets

A total of 87 patients received CHM treatment, and 469 CHM prescriptions were used. These prescriptions contained 257 herbs. Among the 257 types of herbs, the maximum frequency of a single herb was 375. Eighty-nine herbs with use frequency > 30 (375 × 8%) were selected for correlation analysis and defined as commonly used herbs. Twelve of these herbs were closely associated with survival. The other 77 commonly used herbs were applied to relieve the major complications of OvCa, mainly by relieving ascites and diuresis and alleviating abdominal discomfort symptoms. In addition, herbs used with a frequency < 8% were defined as uncommonly used herbs, which were usually applied to relieve uncomfortable symptoms, such as pain, vomiting, fever, dyspepsia, constipation, diarrhea, or insomnia. The statistical results indicated that the 12 herbs we selected were closely related to improved survival (*p* < 0.05, correlation coefficient > 0.27). These 12 herbs were *Radix Aconiti Lateralis Preparata* (RALP), *Cortex Eucommiae* (CE), *Gecko* (G), *Herba Leonuri* (HL), *Rhizoma Zingiberis* (RZ), *Herba Verbenae* (HV), *Semen Vaccariae* (SV), *Herba Taxilli* (HT), *Radix Phytolaccae* (RP), *Radix Notoginseng* (RN), *Ramulus Cinnamomi* (RC), and *Cortex Moutan* (CM).

**Figure 3:**
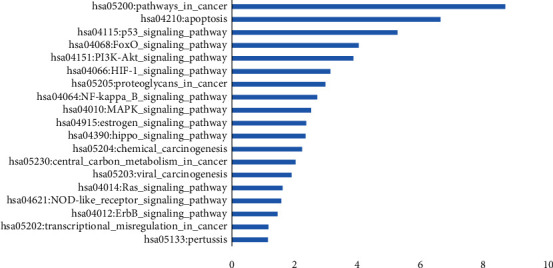
Enrichment analysis of 207 candidate targets. Enrichment analysis showed that candidate targets were frequently involved in intracellular signaling cascades and apoptosis (-Log10 (*p* value)).

Furthermore, the 189 ingredients present in these 12 herbs were suggested to be related to OvCa treatment. To further clarify the potential molecular mechanisms of these herbal medicines, the targets of the proposed active ingredients were identified. These candidate ingredients yielded 207 potential OvCa targets. As shown in Figure 3, the primary targets were TP53, nuclear factor kappa-B (NF-*κ*B), and apoptosis signaling pathways, which are involved in cell proliferation, apoptosis, and metastasis.

### 3.6. Targets Prediction in the Candidate Ingredient-Target Network

**Figure 4:**
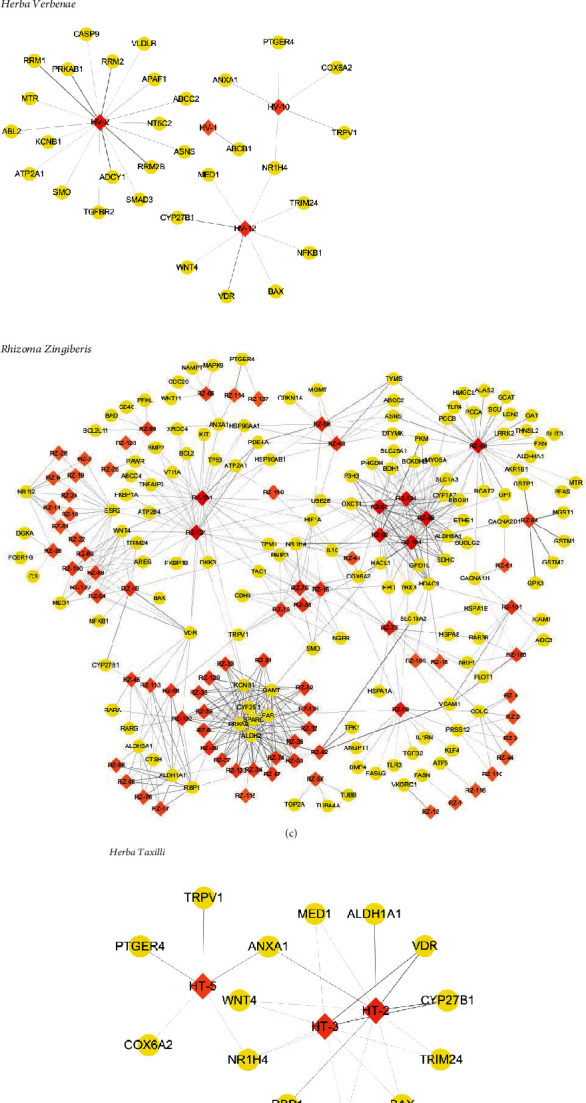
The ingredient-target networks. The diamond nodes represent ingredients; the circular nodes represent targets. The colors of the nodes are illustrated for red to yellow in descending order of degree value.

The 12 herbs contain 381 compounds, 189 of which have an effect on OvCa. Although each drug has a different target, they all act on some common targets. These targets can interact with each other. Ingredient-target networks of each herb are shown in Figure 4. Objects near the edges interact with candidate components much less than those near the center, which also indicates that a single candidate component will affect many candidate targets. In other words, a target can be regulated by not only one component but also multiple components. The major ingredients and major targets of the 12 herbs involved in OvCa treatment are shown in Table 3.

**Figure 5:**
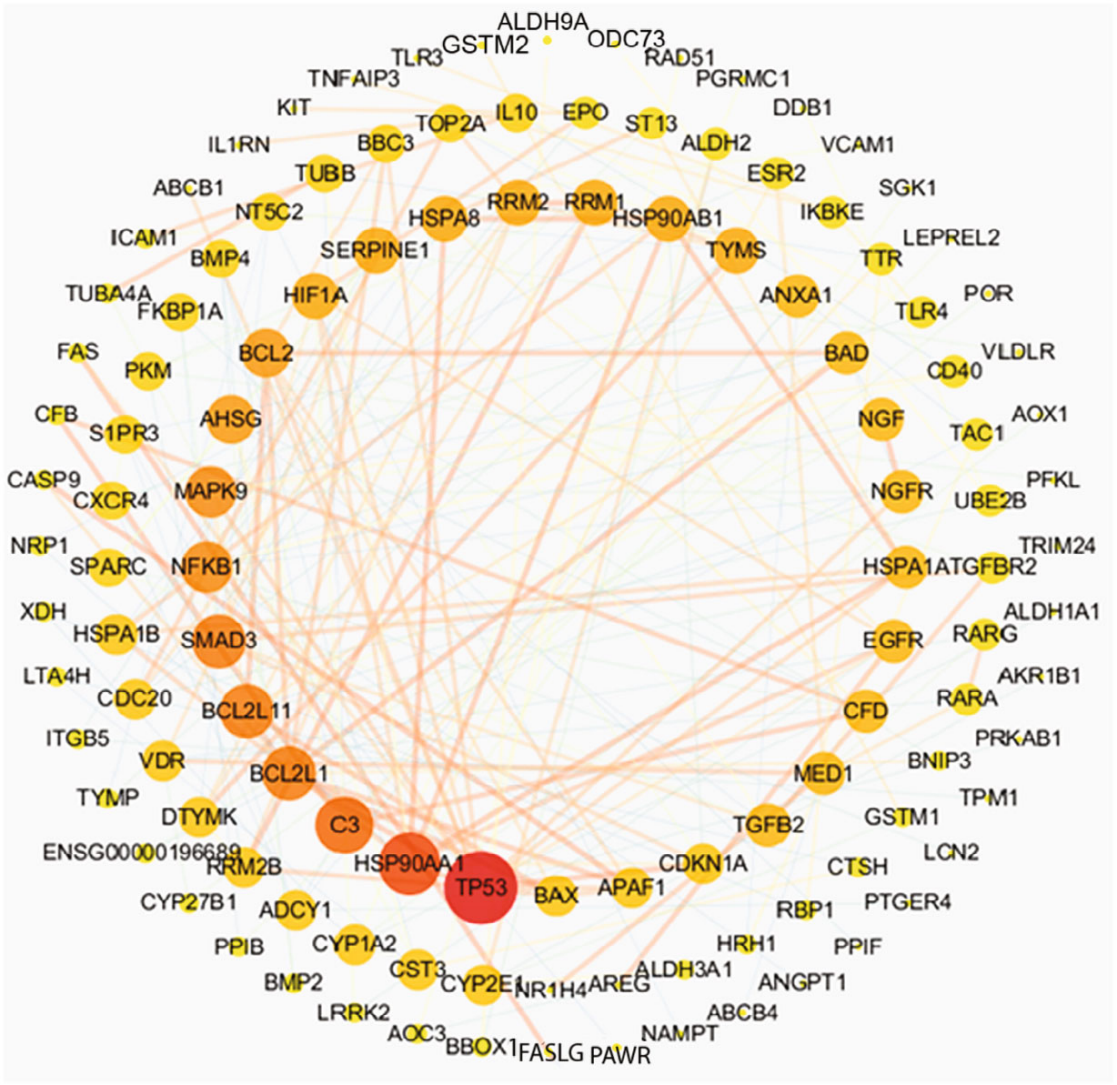
Protein-protein interaction of 207 candidate protein targets. In this network, the big red nodes have a higher degree of freedom, which indicates that these genes may be more important targets in the 12 herbs and the development of OvCa.

We used the String database to establish the protein-protein interactions (PPI) of the 207 candidate protein targets shown in Figure 5 [21]. According to the prediction of the PPI network, we have formed a network of interactions between core proteins whose combined scores are higher than 0.9 [22, 23]. In this network, the big red nodes (TP53, BCL-2, BAX, NF-*κ*B, FAS, VDR, and CDKN1A) have a higher degree of freedom, which indicates that these genes may be more important targets in the 12 herbs and the development of OvCa.

### 3.7. Holistic Mechanisms of Anti-OvCa Medicinal Herbs

Like other cancers, the pathogenesis of OvCa is very complex and is caused by multiple parallel pathways. In this research, 207 tumor herbal-associated proteins involved in tumorigenesis were identified as the targets of 12 herbs using network analysis. We found that TP53, BCL-2, BAX, NF-*κ*B, FAS, VDR, and CDKN1A had the most direct interactions with these herbs, suggesting that these proteins might play important roles in the treatment of OvCa.

Based on the network and multiobjective computing methods, we found that multiple predictive components of the 12 herbs could act on multiple pathogenesis of OvCa. For example, TP53, FAS, CDKN1A, BCL-2, and NF-*κ*B play an important role in apoptosis and antiapoptosis. In terms of cell proliferation, TP53 can be inhibited by a variety of predictive components.

**Figure 6:**
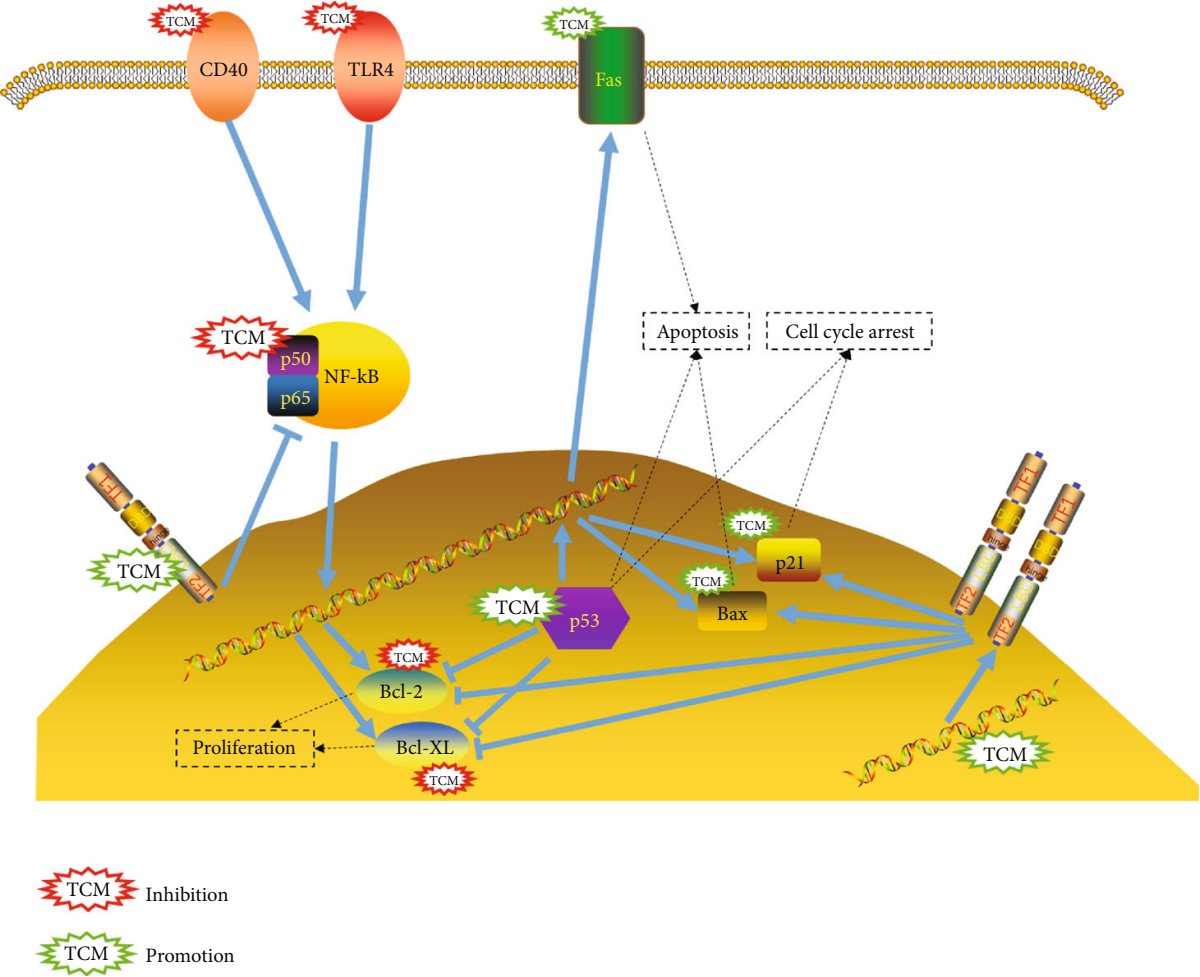
Signaling pathways involved in the actions of the 12 herbs against OvCa. The 12 CHM herbs may exert anti-OvCa activity through (1) the regulation of apoptosis-related protein expression, which promotes OvCa cell apoptosis and inhibits the development of OvCa, (2) the promotion or inhibition of protein expression on the p53 signaling pathway, and (3) the inhibition of the NF-*κ*B signaling pathway by directly inhibiting NF-*κ*B.

In order to better comprehend the target functions associated with the 12 herbs, we mapped their targets to canonical signaling pathways identified in the Kyoto Encyclopedia of Genes and Genomes (KEGG) and summarized the most relevant pathways (Figure 6). The P53 signaling pathway was the strongest pathway. TP53, FAS, BAX, BCL-2, and CDKN1A all played a role in this signaling pathway.

### 3.8. Experimental Validation

**Figure 7:**
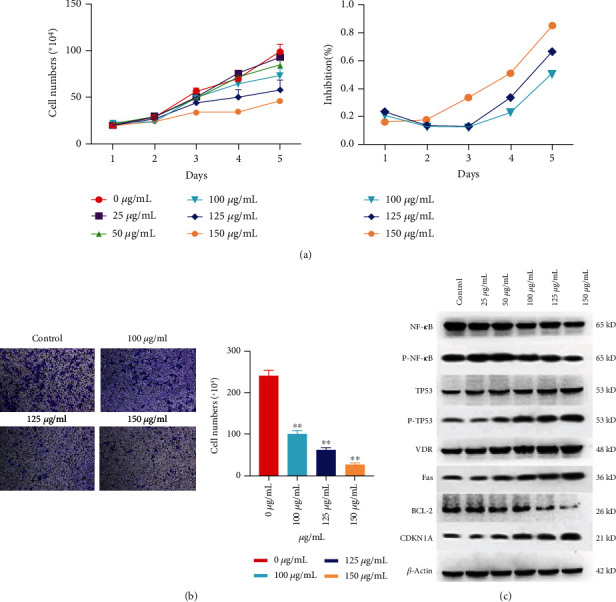
Effect of aqueous extract of 12 herbs on cell proliferation, cell migration, and predicted targets. (a) The statistical views of cell proliferation (left) and cell inhibition rate (right). The 12 herbs had obvious inhibitory effects on cell proliferation after 72 hours at doses of 100 *μ*g/mL, 125 *μ*g/mL, and 150 *μ*g/mL *in vitro*. (b) The result of Transwell. The aqueous extract also significantly suppressed cell migration at 16 hours (^∗∗^*p* < 0.05). (c) Western blot assay analyzed NF-*κ*B, p-NF-*κ*B, TP53, p-TP53, VDR, FAS, BCL-2, and CDK1A, after the treatment of aqueous extract of 12 herbs.

The effectiveness of the 12 herbs, their close relation to survival, and their possible targets were verified by experiments. A trypan blue staining assay and CCK-8 were used to evaluate the inhibition of tumor cell proliferation. The antimigration effects were evaluated using Transwell. The core predicted targets were detected using Western blot.  Figure 7(a) shows the statistical antiproliferation and inhibition rates of the 12 herbs.  Figure 7(b) shows the Transwell results of the antimetastatic effects of the 12 herbs. These experimental results indicated that aqueous extracts of the 12 herbs had obvious inhibitory effects on cell proliferation after 72 hours at doses of 100 *μ*g/mL, 125 *μ*g/mL, and 150 *μ*g/mL *in vitro*. The aqueous extract also significantly suppressed cell migration at 16 hours at doses of 100 *μ*g/mL, 125 *μ*g/mL, and 150 *μ*g/mL. Interestingly, the aqueous extracts of the 12 herbs obviously inhibited cell migration at 16 hours, but only obviously inhibited cell proliferation after 72 hours. It seems that the aqueous extracts affect cell migration after only a short time, but anticell proliferation takes longer. Furthermore, the aqueous extracts affected the expression of VDR, BCL-2, NF-*κ*B, p-NF-*κ*B, TP53, p-TP53, FAS, and CDK1A. As shown in Figure 7(c), the aqueous extracts obviously decreased BCL-2, NF-*κ*B, and p-NF-*κ*B and increased VDR, TP53, p-TP53, FAS, and CDK1A at doses of 100 *μ*g/mL, 125 *μ*g/mL, and 150 *μ*g/mL.

## 4. Discussion

Previous studies have shown that traditional Chinese medicine often plays an antitumor role in cancer treatment by reducing the resistance of patients to chemotherapy drugs, while improving their immunity and nutritional status. With the advancement of molecular biology technologies, more evidence is emerging that certain components in some traditional Chinese medicines can directly affect OvCa pathogenesis. For example, the compound, fuling granule, not only inhibits OvCa cell proliferation *in vitro* but also inhibits TGF-*β*-induced, epithelial-mesenchymal transitions (EMT) [24]. In addition, *Xanthium strumarium* L. extracts can cleave poly (ADP-ribose) polymerase (PARP) [25]. Clinical research data shows that CHM treatment of OvCa likely has similar results. This study verifies these positive results for CHM treatment in patients with OvCa. The median survival time for patients in the CHM group (91 months) was longer than that in the non-CHM group (65 months). For patients with FIOG stage III and FIOG stage IV, the median survival time of the CHM group was significantly extended. The OS rate of the CHM group was significantly higher than that of the non-CHM group (*p* < 0.001). Moreover, the long-term survival rate of patients with OvCa in the CHM group is more optimistic, although CHM's molecular mechanisms in OvCa need to be further elucidated.

Through the analysis of clinical data, a total of 12 herbs were found that significantly improved the survival rate of OvCa patients. We connected the targets of these 12 herbs to the corresponding drug components with 1304 OvCa-related targets and found that 207 targets are related to drugs and diseases at the same time. After enrichment analysis of the targets, we found that these targets included P53, FoxO, NF-*κ*B, PI3K/AKT1, and the apoptosis signaling pathway. These pathways are signaling cascades within cells and are related to the proliferation, apoptosis, immunity, metabolism, and metastasis of cancer cells [26–28]. After PPI network analysis of 207 related targets, VDR, BCL-2, NF-*κ*B, TP53, FAS, and CDKN1A were found to play a primary role in these herbs and diseases. Some of these targets, such as VDR and NF-*κ*B, have previously been shown to be directly related to OvCa [29, 30].

The 12 herbs contain 381 ingredients. Of these, 189 ingredients have a potential impact on OvCa and need further research. Each of the 189 components may correspond to one or more targets, and each target may also correspond to multiple components. Among the 189 kinds of components, stigmasterol, *β*-sitosterol, cetoside, quercetin, oleanolic acid, apigenin, paeoniflorin, artemisinin, and kaempferol, appear more frequently. Quercetin [31, 32], stigmasterol [33, 34], and oleanolic acid [35, 36] have been documented to have antitumor effects.

The Transwell, cck-8, and trypan blue staining tests evaluated the anti-OvCa effect of the 12 herbs. These experimental results showed that their aqueous extracts had significant inhibitory effects on cell proliferation and migration, which dose and time dependent. When the dose of the aqueous solution in vitro reached 100 *μ*g/mL, cell proliferation and migration were significantly inhibited. In addition, the Western blot results confirmed that the aqueous extracts obviously decreased the expression of BCL-2 and NF-*κ*B and increased the expression of VDR, TP53, FAS, and CDKN1A. These results indicate the role of network pharmacology in target prediction.

The proteins expressed by TP53, CDKN1A, BCL2, FAS, and BAX are all related to the p53 signaling pathway [37]. The pathway plays a vital role in the occurrence and development of OvCa and has a regulatory effect on the regulation of the OvCa cell cycle and cell biological behavior [38, 39]. Numerous studies have shown that TP53 has a totipotent status in cancer biology. It participates in coordinating the basic events that cancer initiation and development must overcome and is considered to be a characteristic of cancer [40, 41]. In OvCa, TP53 is closely related to the cell cycle, apoptosis, and microangiogenesis [42]. The expression of TP53 was significantly associated with overall poor survival and CA125 levels in OvCa [43]. The FAS gene is the most important gene for regulating apoptosis. The abnormal FAS/FAS ligand (FASL) pathway is not only related to tumorigenesis and development but also may be related to the sensitivity of tumor cells to some chemotherapy drugs. Increasing the expression levels of the FAS gene in OvCa cells may be an effective method for OvCa gene therapy [44, 45]. BCL-2 is involved in key processes of cells, including preventing S-phase cell cycles and promoting apoptosis. Therefore, interference with BCL-2 expression is an effective strategy for treating cancer [46]. The cell cycle inhibitor CDKN1A can be induced by a p53-dependent mechanism [47]. The induced activated CDKN1A is a protein playing multiple roles in the DNA damage response and is also involved in the regulation of transcription, apoptosis, DNA repair, and cell motility [48].

As one of the gene regulatory factors that control cell proliferation and cell survival, inhibitors of the NF-*κ*B signaling pathway have been proposed as potential therapeutic agents in OvCa [29]. In this study, we found that most predictive components act on VDR. A large number of studies have confirmed that 1*α*, 25-dihydroxyvitamin D3 and its analogs mainly rely on the VDR pathway to regulate cell proliferation and differentiation, induce apoptosis, and inhibit tumor angiogenesis and thus inhibit tumor cell growth [49]. Interestingly, in cell proliferation regulation and apoptosis, VDR can inhibit the development of OvCa by interacting with BCL-2 family proteins. For example, *α*, 25-hydroxyvitamin D3 and VDR can promote apoptosis by inhibiting the expression of the antiapoptotic proteins Bcl-2 and Bcl-XL or by inducing the expression of the proapoptotic proteins BAX, BAK, and BAD [50]. In addition, these targets are mapped to canonical signal pathways identified in KEGG.

Overall, our results indicate that the 12 CHM herbs may exert anti-OvCa activity through (1) the regulation of apoptosis-related protein expression, which promotes OvCa cell apoptosis and inhibits the development of OvCa, (2) the promotion or inhibition of protein expression on the p53 signaling pathway, and (3) the inhibition of the NF-*κ*B signaling pathway by directly inhibiting NF-*κ*B.

Network pharmacology is a commonly used method to measure efficacy and reveal functional mechanisms of multitarget drugs, but it also has limitations. One limitation is that the CHM ingredients selected by DL values and protein scores may not coincide with their exact ingredients. In addition, since the accuracy of the target forecasting tools varies, the results obtained using different forecasting tools may be incompatible. Moreover, the number of clinical cases counted in this study is not large enough. Our use of Spearman's bivariate correlation analysis to obtain strongly correlated herbs for positive results may not accurately reflect the actual clinical situation. In future research, more reliable results could be obtained by collecting a large number of clinical data samples, integrating data from different databases, performing accurate experimental research, and using appropriate statistical methods.

## Figures and Tables

**Table 1:** Patients' baseline characteristics and treatments between CHM group and non-CHM group.

		Non-CHM (*n* = 115)	CHM (*n* = 87)	*p* value
Age	<45	16	21	0.154
	45-59	76	53	
	≥60	23	13	
Tumor location	Left	13	18	0.102
	Right	41	22	
	Bilateral	61	47	
Pathological pattern	Endometrioid adenocarcinoma	31	15	0.263
	Serous adenocarcinoma	74	63	
	Other types	10	9	
FIGO stage	Stage I	7	3	0.365
	Stage II	25	12	
	Stage III	68	58	
	Stage IV	15	14	
CA-125 (kU/L)	<35	3	2	0.971
	35-280	21	15	
	>280	91	70	
Chemotherapy method	Intravenous injection	99	72	0.516
	Intravenous injection+intraperitoneal perfusion	16	15	
Radiotherapy	Yes	6	8	0.270
	No	109	79	
Biotherapy	Yes	24	28	0.069
	No	91	59	
Targeted therapy	Yes	6	4	0.841
	No	109	83	

**Table 2:** Univariate and Multivariate analyses of variables influencing survival of 202 patients with OvCa.

	Univariate analysis		Multivariate analysis			
Characteristics	*N* (%)	*p* value	*β*	Exp (*β*)	95% CI	*p* value
Age (years)		0.028	0.332	1.394	0.984-1.975	0.062
<45	37 (18.3)					
45-59	129 (63.9)					
≥60	36 (17.8)					
Tumor location		<0.001	0.384	1.468	1.083-1.990	0.013
Left	31 (15.3)					
Right	63 (31.2)					
Bilateral	108 (53.5)					
Pathological pattern		0.023	0.044	1.045	0.837-1.303	0.669
Endometrioid adenocarcinoma	46 (22.8)					
Serous adenocarcinoma	137 (67.8)					
Other types	19 (9.4)					
FIGO stage		<0.001	0.841	2.320	1.688-3.188	<0.001
Stage I	10 (5.0)					
Stage II	37 (18.3)					
Stage III	126 (62.4)					
Stage IV	29 (14.4)					
CA-125 (kU/L)		0.009	0.426	1.531	0.888-2.641	0.126
<35	5 (2.5)					
35-280	36 (17.8)					
>280	161 (79.7)					
Chemotherapy method		0.034	0.856	2.353	1.427-3.882	0.001
Intravenous injection	171 (84.7)					
Intravenous injection+intraperitoneal perfusion	31 (15.3)					
Radiotherapy		0.032	1.186	3.273	1.018-10.522	0.047
Yes	14 (6.9)					
No	188 (93.1)					
Biotherapy		0.421	—	—	—	—
Yes	52 (25.7)					
No	150 (74.3)					
Targeted therapy		0.236	—	—	—	—
Yes	10 (5.0)					
No	192 (95.0)					
CHM		0.004	-0.853	0.426	0.279-0.650	<0.001
Yes	87 (43.1)					
No	115 (56.9)					

**Table 3:** The major ingredients and major targets of the 12 herbs involved in OvCa treatment.

Chinese name	Latin name	Major ingredients	Number of targets	Major targets	Average dosage(g)	Correlation coefficient	*p* value
附子	*Radix Aconiti Lateralis Preparata*	Higenamine (RALP-3) P-Aminophenol (RALP-9)	14	EPO FAS FASLG	6	0.313	0.002
杜仲	*Cortex Eucommiae*	Civetone (CE-17) Epiquinidine (CE-23) N-Triacontanol (CH-46)	90	BCL2 TP53 NFKB1 VDR	10	0.353	0.004
蛤蚧	*Gecko*	Glutathion (G-3) Guanine (G-4)	60	BCL2 TP53 VDR	5	0.336	0.006
益母草	*Herba Leonuri*	Guanine (HL-6) Lauric acid (HL-8)	78	BCL2 TP53 VDR	30	0.328	0.008
生姜	*Rhizoma Zingiberis*	Coprine (RZ-49) Shyobunone (RZ-121) Zingiberone (RZ-131)	143	BCL2 TP53 NFKB1 VDR	10	0.327	0.008
马鞭草	*Herba Verbenae*	Adenosine (HV-2)	31	WNT4 NFKB1 BAX VDR	30	0.315	0.011
王不留行	*Semen Vaccariae*	Hydroginkgolinic acid (SV-1) Melanin (SV-3)	39	BCL2 TP53 VDR	30	0.313	0.011
桑寄生	*Herba Taxilli*	Beta-amyrin (HT-2) Oleanolic acid (HT-5)	14	NFKB1 BAX VDR	15	0.307	0.013
商陆	*Radix Phytolaccae*	Esculentic acid (RP-3) Jaligonic acid (RP-15)	9	ANXA1 VDR	9	0.301	0.015
三七	*Radix Notoginseng*	Coprine (RN-15) Heneicosanic acid (RN-44) Hexadecanoic acid (RN-48) Nonadecanoic acid (RN-55) Pentadecanoic acid (RN-69)	118	BCL2 TP53 NFKB1 VDR	2	0.293	0.018
桂枝	*Ramulus Cinnamomi*	Anethole (RC-4) Styrene (RC-20)	59	NFKB1 VDR BCL2L11	10	0.292	0.018
牡丹皮	*Cortex Moutan*	Gallicacid (CM-5) Suffruticosol A (CM-17)	15	WNT4 VDR ESR2	9	0.272	0.028

## Data Availability

The data used to support the findings of this study are included in this article.
